# Empathic Accuracy in Adolescent Girls with Turner Syndrome

**DOI:** 10.1007/s10803-021-05089-3

**Published:** 2021-06-03

**Authors:** M. Klabunde, A. Piccirilli, J. Bruno, M. Gendron, A. L. Reiss

**Affiliations:** 1 Department of Psychology and Centre for Brain Sciences, University of Essex, Wivenhoe Park, C04 3SQ UK; 2 Center for Interdisciplinary Brain Sciences, Department of Psychiatry and Behavioural Sciences, Stanford University School of Medicine, Stanford, CA USA; 3 Department of Psychology, Yale University, New Haven, CT USA; 4 Department of Radiology, Stanford University School of Medicine, Stanford, CA USA; 5 Department of Pediatrics, Stanford University School of Medicine, Stanford, CA USA

**Keywords:** Turner syndrome, Empathy, Theory of mind, Social cognition and neurogenetic disorders

## Abstract

To examine the potential mechanisms underlying social deficits in Turner Syndrome, we administered the empathic accuracy task (EAT) -a naturalistic social cognition task- and a (control) visual-motor line-tracking task to 14 girls with TS was compared to 12 age-matched typically developing girls (TD; ages 12 to 17). Empathic accuracy was compared across positive and negative emotionally valanced videos. We found that TS differs from TD on empathic accuracy ratings for negative videos; no differences were detected for the positive videos or for the control line tracking task. Thus, our findings suggest impaired detection of negatively valanced empathic interactions in TS and may help inform the future development of social-cognition treatment strategies for girls with TS.

Turner syndrome (TS) is a disorder in human females in which part or all of the genetic material from one X-chromosome is absent. Girls with TS display a variety of physical, developmental and cognitive abnormalities. These include cardiovascular problems (Ho et al., 2004), short stature, ovarian failure (Ross et al., 2004) and relative deficits in visual-spatial-motor and executive functions (Mauger et al., 2018; Saenger et al., 2001). Additionally, girls with TS have documented social difficulties and struggle with social anxiety (Hong et al., 2011; McCauley, Feuillan, Kushner, & Ross, 2001; McCauley, Sybert, & Ehrhardt, 1986; J. Ross et al., 2000). Difficulties include problems forming and maintaining social relationships, impaired social competence, and having few close friends (Lagrou et al., 2006; McCauley, Kay, Ito, & Treder, 1987; McCauley, Ross, Kushner, & Cutler Jr., 1995). Girls with TS and their families report that social difficulties become more prominent during adolescence (Wolstencroft, Mandy, & Skuse, 2020). Adolescent girls with TS describe feeling “out of sync” with their peers and express an awareness of their social difficulties during interviews (Wolstencroft et al., 2020) while mutually indicating a desire for social interactions (Wolstencroft & Skuse, 2019). Several recent studies suggest that girls with TS perform poorly on tasks of social cognition, which may contribute to their reported social difficulties. Specifically, memory for faces (Hong et al., 2011), eye gaze (Elgar et al., 2002; Lawrence, Campbell, et al., 2003; Lawrence, Kuntsi, et al., 2003; Mazzola et al., 2006) and affect recognition (Anaki et al., 2016; Good et al., 2003; Hong et al., 2014; Lawrence, Kuntsi, et al., 2003; Mazzola et al., 2006; Roelofs et al., 2015; Ross et al., , 2002, 2004) deficits were found. For affect recognition tasks requiring participants to identify the emotions associated with static pictures of faces, girls with TS demonstrate impaired recognition for negative emotions, yet intact recognition for positive emotions (Hong et al., 2014; Lawrence, Kuntsi, et al., 2003; Mazzola et al., 2006; McCauley et al., 1987; Skuse, 2005). Deficits were particularly pronounced for perceiving targets portraying fear (Anaki et al., 2016; Hong et al., 2014; Lawrence, Kuntsi, et al., 2003; Mazzola et al., 2006; McCauley et al., 1987; Skuse, 2005). Affect identification deficits were also present for targets displaying the emotions sadness and disgust (Anaki et al., 2016). Mechanisms underlying these aforementioned affect recognition and social deficits are not yet well understood.

One theory attempting to describe the nature of the social deficits seen in girls with TS suggests that theory of mind (TOM) difficulties may be present (Anaki et al., 2016; Hong et al., 2011; Lawrence et al., 2007) and could be underlying their social problems (Lepage et al., 2014). TOM describes “the ability to reflect on the contents of both one’s own and other’s minds” (p. 3; Baron-Cohen, 2001). Researchers hypothesize that TOM deficits in TS may be influenced by dysfunction in circuitry involved in mirroring (Lepage et al., 2014), affect (Burnett et al., 2010), visual spatial processing (Anaki et al., , 2016, 2018), and processing of eye-gaze cues (Burnett et al., 2010; Lawrence, Campbell, et al., 2003). Despite some initial evidence for the previously mentioned hypotheses, no definitive conclusions about the nature of the TOM deficits in girls with TS can be made.

Previous TOM studies in TS have only used deconstructed and simplified social stimuli, which lack ecological validity. Social interactions, however, are complex and successful social interactions require a well-balanced synthesis of various abilities and behaviours (Zaki & Ochsner, 2012). Therefore, it is necessary to study TOM in TS with naturalistic stimuli in order to fully capture the extent of the subtle yet potentially impactful social deficits in girls with TS (Burnett et al., 2010). For this study, we implemented an empathic accuracy task (EAT) in adolescent girls with and without TS. Empathic accuracy tasks attempt to simulate naturalistic social interactions by having study participants watch a video of a real person (i.e., not an actor) telling an emotionally laden personal story. Participants are then asked to rate or describe the internal mental states of that target. In the version used here, participants continuously rated the emotional valence felt by a target in a video; both the participant and the target person provide continuous ratings of the perceived emotional valence. These ratings are compared and provide a measure of empathic accuracy, based on the assumption that the target can report on their own affective state with high fidelity.

Because one hypothesis suggests that visual-spatial deficits in girls with TS may specifically influence their affect recognition abilities (Anaki et al., , 2016, 2018) and since girls with TS are also at risk for visuomotor deficits, we administered a vertical line tracking task as a control for the EAT. This control task was successfully used in a previous study of persons with schizophrenia (Lee et al., 2011), a condition also characterized by relative deficits in motor, attention and visual spatial processing. Here, we hypothesized that girls with TS would demonstrate relative deficits in empathic accuracy for negatively-valanced videos that would exceed any potential deficits in line tracking (visuo-motor-attention-processing speed) accuracy. Furthermore, a recent neuroimaging study demonstrated that empathy develops during adolescence in conjunction with the prefrontal cortex (Kral et al., 2017). Although some girls with TS experience spontaneous puberty, it is often delayed or stalled, thus necessitating hormonal intervention with estrogen (Gravholt et al., 2019). Therefore, in order to address the potential influences of pubertal stage on brain and empathy development in girls with TS, participants’ age and/or pubertal development were controlled across groups in this study.

## Methods

### Participants

Fourteen girls with TS and 12 typically developing girls (TD), ages 12 through 17 years of age were recruited from a larger study, which examined longitudinal brain changes associated with TS. Participants with TS were recruited nationally and TD participants were recruited from the San Francisco Bay area. Inclusion criteria for TS included genetic confirmation of Turner syndrome with a 45X karyotype. Controls with developmental delays and significant medical and/or psychiatric illness were excluded from participating in the study. Participant pubertal stage was assessed via self-report on the Tanner scale which includes ratings of breast and pubic hair development (Marshall & Tanner, 1969) and was confirmed by study physicians. Intelligence was measured using the Weschler Abbreviated Scale of Intelligence (WASI; Wechsler, 1999)). Additional recruitment and screening details can be found in a previous paper from our group (Green et al., 2017). The Stanford University Institutional Review Board (IRB) approved the study and the study was conducted in line with the IRB’s standards. All participants assented and their parents consented prior to participating in the study.

### Empathic Accuracy Task

We adapted a version of the empathic accuracy task (EAT) for adolescents and administered it to all study participants. For the EAT, we selected our videos from a pool of stimuli and choose videos of late-adolescent female volunteers ages 18–19, who appeared younger than their age. Videos were between 90 and 150 s in duration and were presented as mp4 files embedded within the task stimuli, which were presented to participants by Psychtoolbox via MATLAB R2017b. We chose three positive and three negative videos that included topics applicable to adolescents. Positive video topics included telling a boyfriend or girlfriend “I love you” for the first time (Video 1), excelling at a sports competition (Video 3), and spending quality time with one’s family (Video 6). Negative video topics included death of a pet (Video 2), moving away from home for the first time (Video 4), and breaking up with a romantic partner (Video 5). We only presented videos of female volunteers since we were interested in learning how empathic accuracy may influence social relationships in girls with TS. Age related preferences for friends occur in girls, such that younger teen girls primarily have same sex friendships (85% of best friends are of the same sex in grades six through seven). With age, adolescents form more opposite sex friendships (e.g., 79% of best friends are of the same sex in eight grade for girls; Arndorfer & Stormshak, 2008). The persons in our videos appeared to be of Caucasian, Asian and Pacific Islander ethnicities, which approximated the demographics of our participants.

**Fig. 1:**
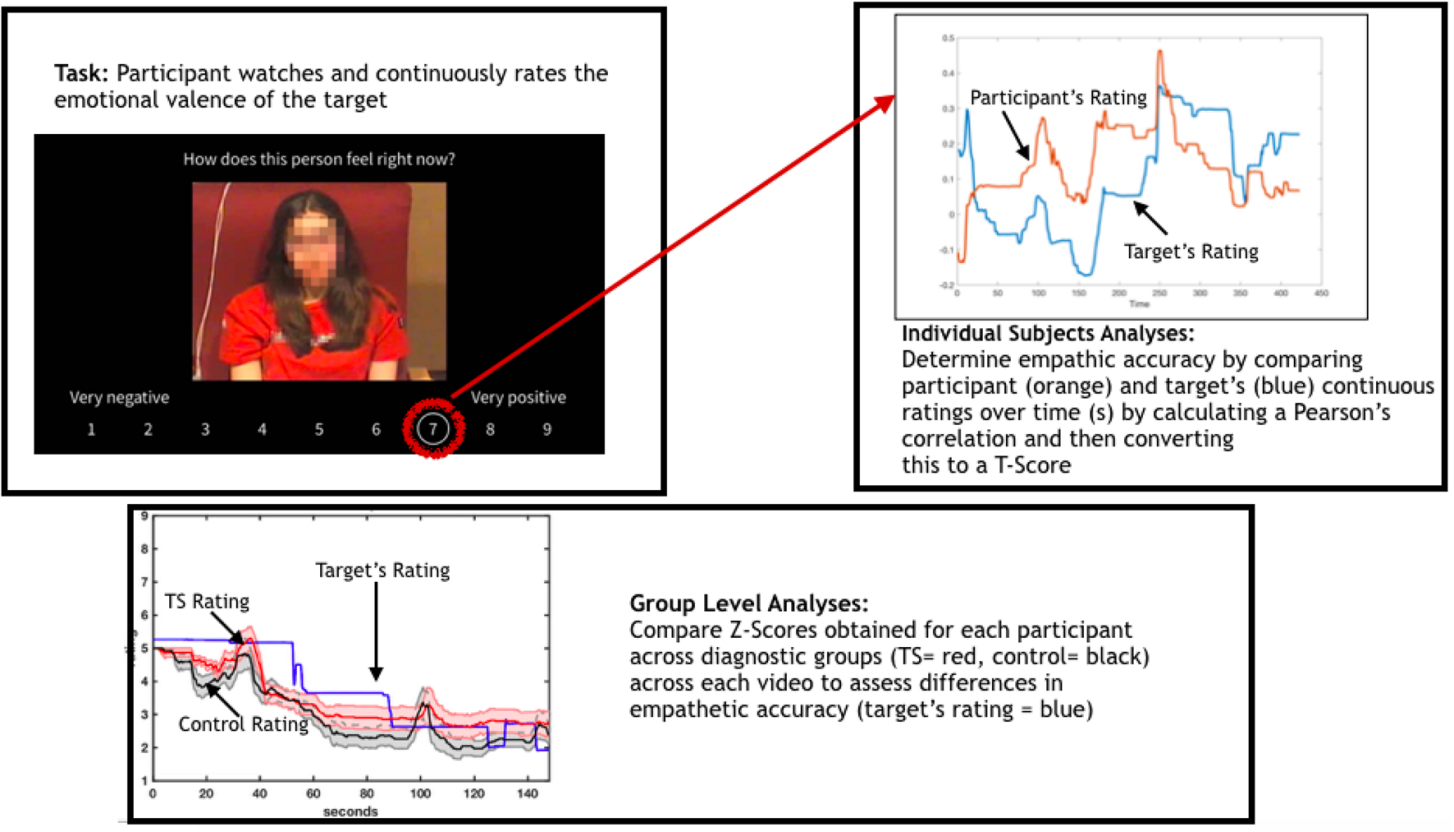
Empathic task and analysis procedures

Procedures for creating empathic videos were similar to previous studies (See Fig. 1) and stimuli were produced by Northeastern University (Zaki et al., 2009). Volunteers (not actors; also called targets) were asked to describe an emotional autobiographical event (either positive or negative) while being filmed and without knowing that their data was going to be included as stimuli within a research study. Then, each target watched and continuously rated the valence (degree of positivity vs negativity) of the emotions felt while describing the event with a dial using a nine-point rating scale (1: very negative, 5: neutral, and 9: very positive). Target volunteers provided consent for the videos to be used for future research. Participants in the present study watched and continuously rated the valence of the target’s feelings in the videos using the right and left arrow keys on a keyboard based on the same nine-point rating scale described above.

### Visual-Motor Control

The visual-motor control condition mimics the visual-motor, attention and processing speed demands of the empathy condition. Participants were shown two trials of a thin vertical line that randomly moved back and forth across the monitor for 90-s. Participants continuously rated the line’s location throughout each 90-s trial using a nine-point visual analogue scale identical to the EAT, and displayed below the video (1: far left, 5: middle, and 9: far right) using the right and left arrow keys on a keyboard. One line trial was shown prior to the six empathy videos and a second line trial was shown after the empathy videos.

### Empathic Accuracy Behavioural Analysis

MATLAB R2017b was used to perform time series downsampling and calculations. Continuous empathy ratings obtained from participants were downsampled from 30 Hz to match the 10 Hz sampling frequency of the target’s ratings. Onsets for the participant and target ratings were aligned. Statistical procedures included calculating empathic accuracy by obtaining the Pearson’s correlation between the participant and targets’ continuous ratings for each task. Fisher’s r to z conversion was then used to convert correlation coefficients to z-scores (See Fig. 1). Similar procedures to previous EAT studies were followed for calculating empathic accuracy ratings in order to maintain fidelity with previously reported results in other populations (Kral et al., 2017; Lee et al., 2011; Mackes et al., 2018; Zaki et al., 2009). Video 1 demonstrated very poor agreement for all participants and therefore was excluded from subsequent analyses (Video 1 average r = 0.01, SD = 0.14; Video 2 average r = 0.72, SD = 0.26; Video 3 average r = 0.41, SD = 0.40; Video 4 average r = 0.69, SD = 0.29; Video 5 average r = 0.74, SD = 0.12; Video 6 average r = 0.70, SD = 0.16). Video 3 demonstrated lower accuracy levels than videos 2,4,5 and 6, though was retained for further analysis since empathic accuracy was not at floor (as in Video 1). The average emotion valence ratings and standard deviations for the target’s ratings were as follows: Video 1 mean = 6.83, SD = 1.50, Video 2 mean = 4.86, SD = 0.91, Video 3 mean = 6.81, SD = 0.92, Video 4 mean = 3.77, SD = 0.18, Video 5 mean = 3.68, SD = 0.64; Video 6 mean = 7.29, SD = 0.80.

To assess whether developmental stage and intelligence (Full scale IQ (FIQ), Verbal Comprehension Index (VCI) and Perceptual Reasoning Index (PRI) should be included as a covariate within our primary analyses, separate Pearson correlations were used to examine the relationships between empathic accuracy (z-scores) with age and IQ scores. Separate Spearman’s rho correlations were used to examine the relationship between the empathic accuracy (z-scores) and the participant’s average Tanner score (the average of Tanner breast and pubic hair ratings were obtained). Significance across these correlations was determined by a Bonferroni correction, which was applied to control for multiple tests for developmental stage (age and average Tanner, *p* = 0.005) and intelligence (IQ, VCI and PRI, *p* < 0.003) separately. To examine differences in group performance on each of the videos, we conducted a one-way MANOVA that included empathic accuracy z-scores for Videos 2–6 as dependent variables.

### Visual-Motor Behavioural Analyses

MATLAB was used to conduct line tracking analyses in a manner similar to the empathic accuracy analysis. Line tracking data were sampled at 10 Hz and line tracking accuracy was calculated by conducting a Pearson’s correlation between the participant’s ratings and the values corresponding to the absolute position of the line on the screen. The Fisher’s r to z conversion was used and z-scores were compared between the TS and control groups using a mixed between-within group ANOVA; within groups included the two line trials.

## Results

### Empathic Accuracy Behavioural Results

**Fig. 2:**
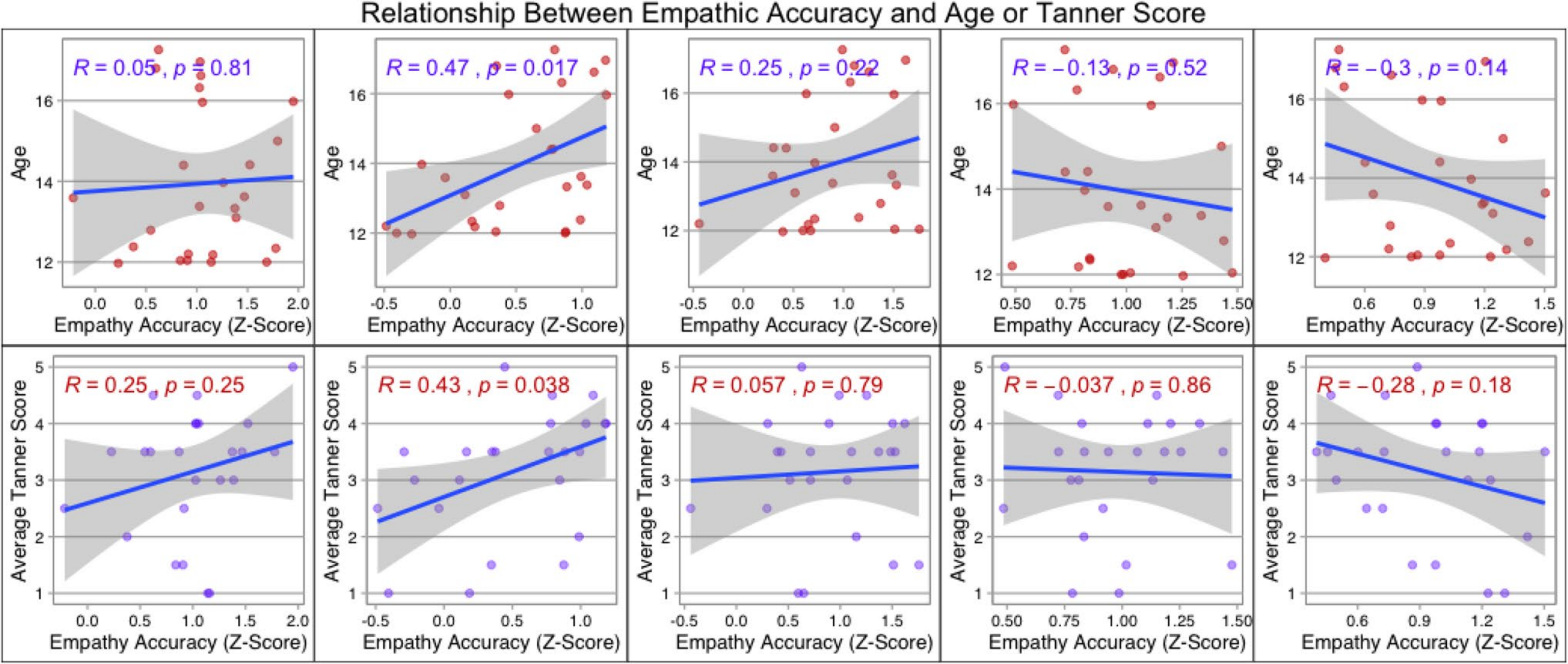
Associations between empathic accuracy and age or average Tanner stage score. Empathic accuracy across both groups were correlated with the participant’s age and average Tanner scores (average of Tanner breast and public hair ratings) for videos 2–6 (displayed left to right). Top rows are correlations with age and the bottom rows display correlations with Tanner Scale

The average r value for all participants on the empathic accuracy task prior to z-transformation was 0.65. (SD = 0.29). A one way ANOVA revealed no significant differences between the two groups in age (F(1,24) = 1.97, *p* = 0.17; TS mean = 14.41, SD = 1.98, TD mean = 13.41, SD = 1.54) and a Mann Whitney U test revealed no significant differences in groups on average Tanner stage (sum between Tanner breast and pubic hair development scores; Mann- Whitney U *p* = 0.44; See Table 1 for demographic information). Significant differences for group were found for intelligence (FSIQ: (F (1,23) = 16.86, *p* = 0.00, VCI: F (1,23) = 10.54, *p* = 0.00), PIC: F (1,23) = 11.63, *p* = 0.00); See Table 1). No significant association between age or Tanner score and empathic accuracy z-scores for individual videos was detected at a Bonferroni corrected value (*p* < 0.005). However, uncorrected p-values of *p* < 0.05 were observed for Video 3 for both age (r = 0.46, n = 24, *p* = 0.02) and Tanner score (rho = 0.52, n = 24, *p* = *0.0*1) (See Fig. 2). No significant associations between IQ (FSIQ, VCI and PSI) and empathic accuracy z-scores were detected for any of the videos at a Bonferroni corrected level (*p* > 0.003).

**Table 1:** Demographic data for each participant

Age	Diagnosis	VCI	PRI	FSIQ	Average tanner stage
12.04	Control	124	109	120	1.5
11.97	Control	108	78	97	3.5
12.79	Control	112	103	111	3.5
13.38	Control	126	103	121	4
12.00	Control	128	109	120	1
13.1	Control	130	131	132	3
14.41	Control	132	112	122	4
12.34	Control	114	115	122	3.5
15.96	Control	130	83	107	4
15.98	Control	114	100	105	5
12.00	Control	N/A	N/A	N/A	N/A
15.00	Control	N/A	N/A	N/A	N/A
17.26	Turner	96	98	84	4.5
12.04	Turner	116	106	112	1.5
16.62	Turner	119	97	114	4.5
16.32	Turner	100	85	102	3
12.2	Turner	102	83	89	2.5
13.97	Turner	108	85	93	3
14.4	Turner	116	65	87	3.5
12.18	Turner	98	73	75	1
16.96	Turner	77	68	84	4
13.62	Turner	98	53	80	3.5
12.38	Turner	100	80	90	2
16.8	Turner	112	78	94	3.5
13.59	Turner	100	91	111	2.5
13.33	Turner	136	100	114	3.5

Average Tanner Stage = average of breast and pubic hair ratings

*VCI* Verbal Comprehension Index on the WISC-IV, *PRI* Perceptual Reasoning Index on the WISC-IV, *FSIQ* Full Scale IQ as measured by the WISC-IV

**Fig. 3:**
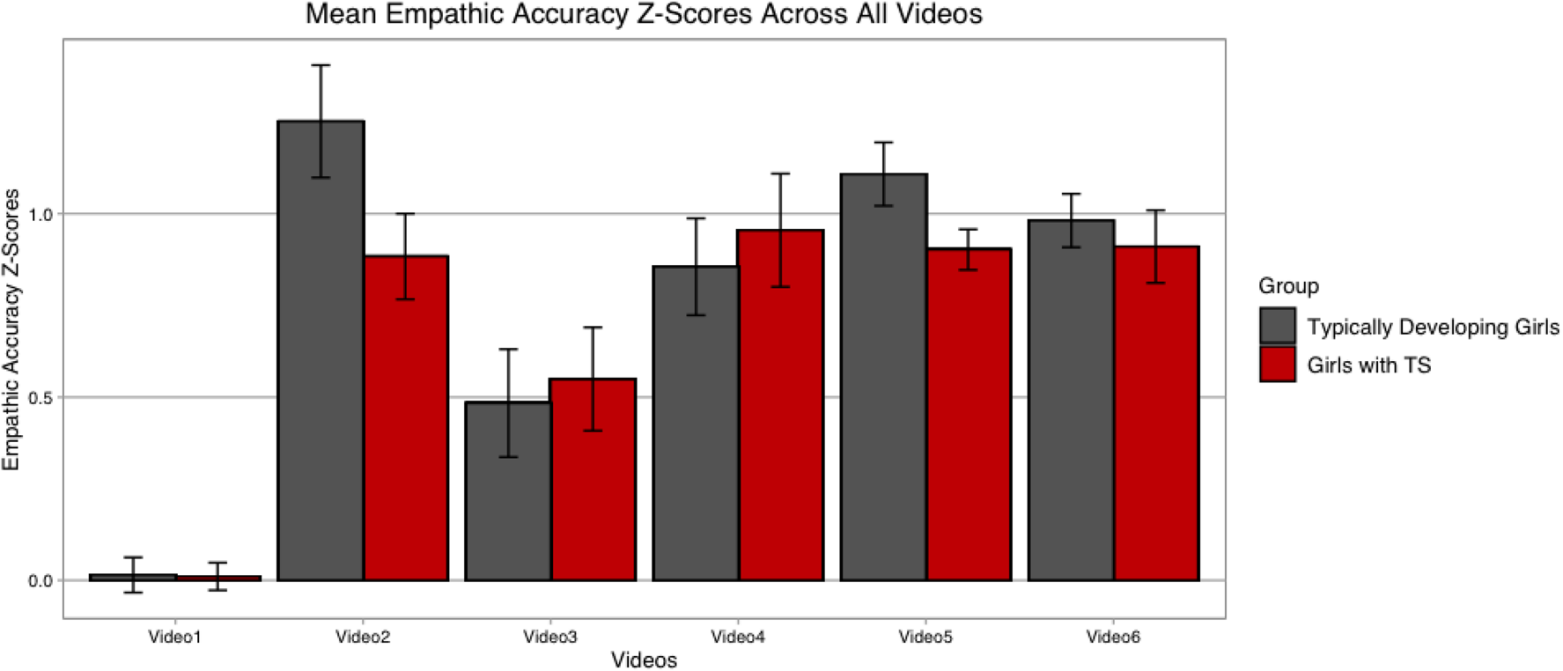
Mean empathic accuracy Z-scores for each video. Red = Girls with TS. Grey = Typically Developing Girls. Video 2 = Death of a pet. Video 3 = Excelling at a sports competition. Video 4 = Moving away from home for the first time. Video 5 = Breaking up with a romantic partner. Video 6 = Spending quality time with one’s family

**Fig. 4:**
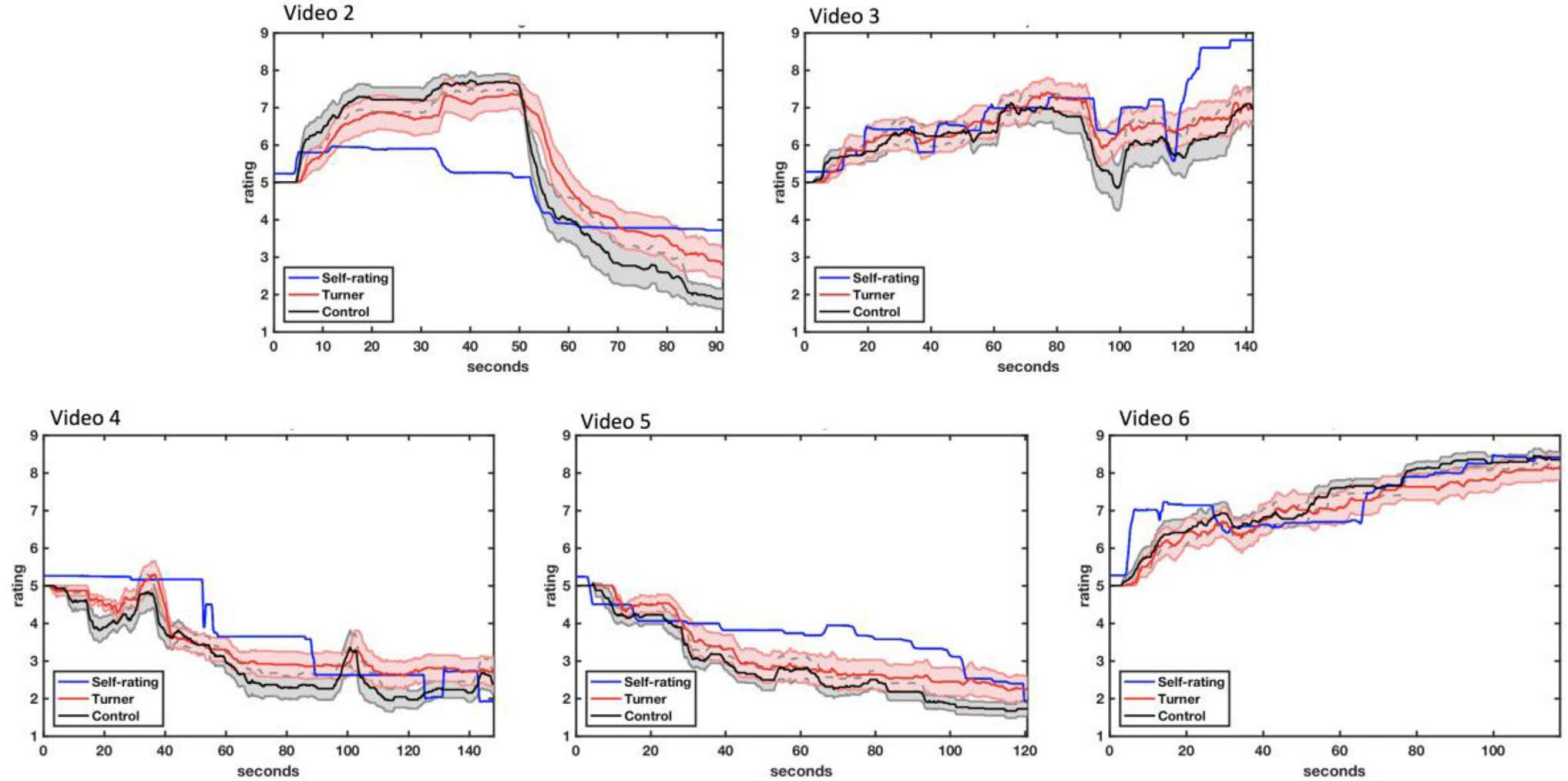
Continuous ratings of the target’s perceived emotional valance. Blue Line = Continues rating of emotional valence by the target. Black line = Mean continuous ratings by control girls. Red line = Mean continuous ratings by the girls with Turner syndrome. Ratings were within a 1 to 9-point scale. Time was measured in seconds. Video 2 = Death of a pet. Video 3 = Excelling at a sports competition. Video 4 = Moving away from home for the first time. Video 5 = Breaking up with a romantic partner. Video 6 = Spending quality time with one’s family

Figure 3 demonstrates empathic accuracy z-scores for all six videos and Fig. 4 shows a plot of the target’s continuous ratings in addition to the continuous ratings for the TS and TD groups separately. When examining differences in empathic accuracy across all of the videos, which were specified as dependent variables, our omnibus analyses revealed significant differences for diagnostic group across all videos (F(1,24) = 4.29, p = 0.008, partial η^2^ = 0.52). Between group effects revealed a significant difference for negative Video 5 (F(1,24) = 4.26, *p* = 0.05, partial η^2^ = 0.15, TS mean = 0.90, SD = 0.21, TD mean = 1.11, SD = 0.30) and a trend towards significance and a medium effect size for negative Video 2 (F(1,24) = 3.77, *p* = 0.064, partial η^2^ = 0.14, mean TS = 0.88, SD = 0.43, TD mean = 1.25, SD = 0.53). Significant between group differences were not found for positive Video 3 (F(1,24) = 0.10, *p* = 0.75, partial η^2 =^ 0.04, TS mean = 0.54, SD = 0.53, TD mean = 0.48, SD = 0.51), negative Video 4 (F(1,24) = 0.23, *p* = 0.64, partial η^2^ = 0.01, TS mean = 0.95, SD = 0.58, TD mean = 0.85, SD = 0.46) or positive Video 6 (F(1,24) = 0.31, *p* = 0.58, TS = 0.91, SD = 0.32, TD mean = 0.98, SD = 0.25).

### Line Tracking Behavioural Results

**Fig. 5:**
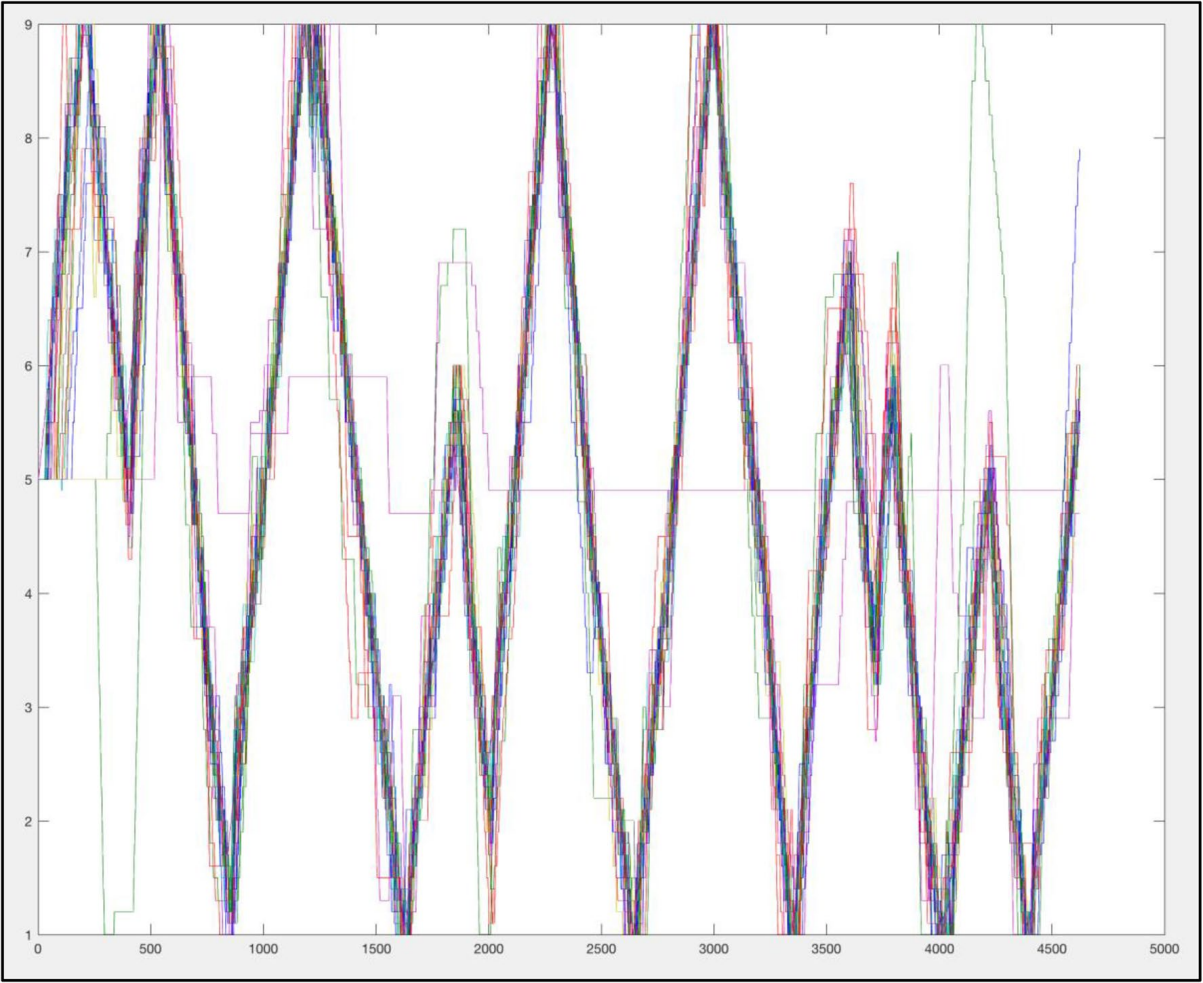
Figure of continuous line tracking data superimposed from all participants. Y axis = rating score for the line position on a 1 to 9 rating scale. X axis = the time axis for the 90 s task at a rate of 50 per second. This figure demonstrates each participant’s line tracking continuous ratings from across both TS and TD groups superimposed upon each other, demonstrating high accuracy across participants and across groups. One participant who’s data displayed via this figure is not consistent with the data from other participants (relatively flat purple line), suggesting a lack of participation in the trial. This participants’ data was removed from the study

Data were excluded from one TS participant who refused to complete the line-tracking task (See Fig. 5). When examining group differences in empathic accuracy across both of the line tasks, our Hotelling’s Trace omnibus F revealed no significant differences (F(1, 22) = 0.93, *p* = 0.41, partial η^2^ = 0.08; Line 1 TS mean = 2.23, SD = 4.2; Line 1 TD mean = 2.49, SD = 3.8; Line 2 TS mean = 2.10, SD = 1.00; Line 2 TD mean = 2.40, SD = 0.77).

## Discussion

We found evidence supporting our hypotheses that girls with TS differ from TD girls on empathic accuracy. Specifically, girls with TS as compared to TD controls demonstrated significantly lower empathy accuracy for the negative video about the death of the target’s dog, a trend towards lower accuracy for the negative video about moving away from one’s home for the first time. Though significant differences were not observed for the negative video about experiencing a breakup, girls with TS performed slightly better in comparison to TD controls. The positive video about being told “I love you” for the first time was excluded due to poor agreement across all participants. For the remaining positive videos, no group differences were found. However, for the positive video about excelling at a sports competition, girls with TS performed slightly better than TD controls and for the positive video about spending quality time with one’s family, TS participants performed slightly worse than the TD control participants (See Fig. 3). Therefore, our results suggest that empathic accuracy abilities in girls with TS vary and may be associated with the emotional valence of the target’s story.

Our results showing lower empathic accuracy for two of our three negatively valanced videos is consistent with the extant literature, which demonstrates impaired affect recognition for negative emotions in TS (Anaki et al., 2016; Hong et al., 2014; Lawrence, Kuntsi, et al., 2003; Mazzola et al., 2006; McCauley et al., 1987; Skuse, 2005). Therefore, socio-emotional deficits in TS may extend beyond simple affect recognition difficulties as measured by decontextualized stimuli to indicating a larger disruption of social cognition in this genetically defined population. Such a phenomenon may explain the subtle yet potentially impactful social deficits and resulting social anxiety reported by adolescent girls with TS in everyday life.

Regarding the theory that emotion processing deficits in TS are influenced by visual-spatial deficits (Lawrence, Kuntsi, et al., 2003; Skuse et al., 2005), we found intact abilities on a moving line-position tracking task, a task which taps into visual-spatial tracking abilities, working memory and processing speed (See Fig. 5). Thus, empathic accuracy deficits observer in this study are unlikely due to tracking abilities, attention problems, processing speed or difficulties with rating abilities for our participants. This control task, however, may not have tapped into the primary visual-spatial deficits impacting TS. Previous studies suggest that girls with TS particularly struggle to perceive and process visual details within a larger global-visual context (Mazzocco et al., 2006), which is also called figure-ground processing. Girls with TS also tend to process facial features in a piecemeal fashion (Anaki et al., 2016). This tendency could be affectively driven for the processing of faces and/or by poor executive functioning abilities rather than being primarily driven by visual factors.

Emotional reactivity within the amygdala impacts fear processing and reduces one’s tendency to examine the eyes for emotional context. This may contribute to affect recognition difficulties, contributing to empathy problems in TS since the eyes are the primary feature for examining negative emotions such as fear (Adolphs et al., 2005; Benuzzi et al., 2007). Abnormal emotional reactivity is present in girls with TS (Hong et al., 2014), thus fear processing deficits in TS may be affectively driven and could explain why girls with TS may spend more time examining non-eye facial features than controls according to eye tracking studies (Lawrence, Campbell, et al., 2003; Lawrence, Kuntsi, et al., 2003; Mazzola et al., 2006). This could influence empathy more so than visual spatial deficits in TS and thus, should be further examined. Furthermore, studies demonstrate a link between emotion reactivity/regulation and executive functioning skills (Drevets & Raichle, 1998). Since girls with TS demonstrate poor executive functioning (Mauger et al., 2018), this may further contribute to deficits in emotion processing and empathy accuracy within this population. Girls with TS could be increasingly recruiting cortical (prefrontal) regions associated with executive functioning during emotional activation. Also, because executive functional skills develop in association with puberty (Stoica et al., 2019), it may also indicate why social deficits in girls with TS appear to be magnified during adolescence (Wolstencroft et al., 2020). To further assess the potential influences of emotion processing and executive functioning on empathic accuracy in TS, future studies should directly and separately examine these topics and their interactions.

Across all participants, our findings suggest higher accuracy when rating the emotional valence of the negative videos (mean = 0.99, SD = 0.06) than the positive videos (mean = 0.73, SD = 0.06). These results differ from a recent study in which participants rated empathic accuracy according to the video’s degree of emotional intensity (rating emotional arousal level) instead of valence; our study only examined emotional valence but not emotional intensity (Mackes et al., 2018). These divergent findings are likely due to the different ratings schemes (rating intensity vs. valence). In particular, the Mackes et al. study implemented stimuli that depicted only one emotion for each video; videos also varied in emotional intensity. Generally, our negative videos conveyed themes pertaining to interpersonal loss and our positive videos portrayed multiple feelings including love, security, pride, and comfort. Emotional intensity was not controlled across our administered videos and, therefore, could further influence empathy accuracy in TS.

The results of our study are important since studying empathic accuracy in girls with TS improves our understanding of the mechanisms underlying social deficits. It highlights that social deficits remain present when using stimuli with improved ecological validity in comparison to the use of more traditional measures (i.e. decontextualized emotional face stimuli and self-report measures). Furthermore, they suggest that empathy accuracy differences could be associated with the emotional valence conveyed within the videos, although these findings are not definitive. Nonetheless, our empathy accuracy results in TS may contribute to the development of treatments aimed at improving social-emotional function in affected girls, such as the development of targeted empathy training. For example, if definitive deficits in empathy accuracy for negatively valanced stories is found in TS, then treatments aimed at helping girls with TS process their own negative emotions could be tested in order to determine whether this change in self emotion processing will impact empathy for negative emotions in another.

### Limitations

Our study has several limitations. First, our sample size was small and we implemented a cross sectional design. Future studies should examine the impact of development by examining empathic accuracy longitudinally with larger samples. More study is needed in order to decipher the full extent of empathic deficits present in TS. Additionally, to further examine the full extent of empathy deficits in TS, the task should be pre-experimented to examine not only the valence but also the arousal, intensity and larger context of the stimuli. Results may be additionally influenced by the individual characteristics of study participants such as whether the participant can relate to the situation conveyed within the video and the participant’s own attachment style. They could also be influenced by the individual characteristics of the targets, such as the target’s sex, race, class and ethnicity, which would be important to examine in future studies. Specifically, one weakness of this study includes the potential generalisability of our stimuli to adolescent girls with TS. We chose to present videos of young looking targets who are just above 18 since they were old enough to consent to the use of their videos as research stimuli. However, because girls with TS tend to have friends who are either their age or younger, this may decrease the validity of how empathy influences friendships in girls with TS. Thus, future studies should examine empathy accuracy in girls with and without same age target stimuli. Moreover, another study weakness includes our limited range and number of stimuli, which should be increased for future replications of this study. We included only six videos, one of which was excluded due to poor agreement while another video was included despite it indicating moderate agreement. Therefore, further replication of this study should include a larger range of videos with good agreement.

## Conclusions

This is the first known study to examine empathic accuracy in girls with Turner syndrome as compared to age-matched controls. As hypothesized, we found between-group differences in empathic accuracy. Group differences did not appear to be due to visual-spatial deficits involved in rating the task, as demonstrated by intact accuracy for both groups on a visual moving line tracking task. Thus, our results suggest that the previously detected deficits for negative emotions may influence empathic accuracy abilities in TS during negatively valanced empathic interactions. However, more research is needed in order to identify the mechanisms underlying our findings. These deficits may help explain social cognition deficits and social anxiety in TS and could contribute to the development of novel treatments for the social deficits present in girls with this condition.
